# Characteristics of disease progress in patients with coronavirus disease 2019 in Wuhan, China

**DOI:** 10.1017/S0950268820000977

**Published:** 2020-05-06

**Authors:** Mengyao Ji, Lei Yuan, Wei Shen, Junwei Lv, Yong Li, Ming Li, Xuefang Lu, Lanhua Hu, Weiguo Dong

**Affiliations:** 1 Department of Gastroenterology, Renmin Hospital of Wuhan University, Wuhan, Hubei, China; 2 Department of Information Center, Renmin Hospital of Wuhan University, Wuhan, Hubei, China; 3 Shanghai Key Laboratory of Artificial Intelligence for Medical Image and Knowledge Graph; 4 Department of Radiology, Suining Central Hospital, Suining, Sichuan, China; 5 Department of Radiology, Renmin Hospital of Wuhan University, Wuhan, Hubei, China

**Keywords:** Clinical stage, COVID-19, Disease progress, SARS-CoV-2

## Abstract

Coronavirus disease 2019 (COVID-19) patients were classified into four clinical stages (uncomplicated illness, mild, severe and critical pneumonia) depending on disease severity. We aim to investigate the corresponding clinical, radiological and laboratory characteristics between different clinical stages. A retrospective, single-centre study of 101 confirmed patients with COVID-19 at Renmin Hospital of Wuhan University from 2 January to 28 January 2020 was enrolled; follow-up endpoint was on 8 February 2020. Clinical data were collected and compared during the course of illness. The median age of the 101 patients was 51.0 years and 33.6% were medical staff. Fever (68%), cough (50%) and fatigue (23%) are the most common symptoms. About 26% patients underwent the mechanical ventilation and 98% patients were treated with antibiotics. Thirty-seven per cent patients were cured and 11 died. On admission, the number of patients with uncomplicated illness, mild, severe and critical pneumonia were 2 [2%], 86 [85%], 11 [11%] and 2 [2%]. Forty-four of the 86 mild pneumonia progressed to severe illness within 4 days, with nine patients worsened due to critical pneumonia within 4 days. Two of the 11 severe patients improved to mild condition while three others deteriorated. Significant differences were observed among groups of different clinical stages in numbers of influenced pulmonary segments (6 *vs.* 12 *vs.* 17, *P* < 0.001). A significantly upward trend was witnessed in ground-glass opacities overlapped with striped shadows (33% *vs.* 42% *vs.* 55% *vs.* 80%, *P* < 0.001), while pure ground-glass opacities gradually decreased as disease progressed (45% *vs.* 35% *vs.* 24% *vs.* 13%, *P* < 0.001) within 12 days. Lymphocytes, prealbumin and albumin showed a downtrend as disease progressed from mild to severe or critical condition, an uptrend was found in white blood cells, C-reactive protein, neutrophils and lactate dehydrogenase. The proportions of serum amyloid *A* > 300 mg/l in mild, severe and critical conditions were 18%, 46% and 71%, respectively.

## Introduction

As the first cases of pneumonia of unknown origin were officially reported in China on 8 December 2019, this disease has rapidly spread to many other regions within China and abroad [1]. As of 13 April 2020, over 1 200 000 confirmed COVID-19 patients have been documented worldwide.

There are many similarities, indicated by the published literatures, between severe acute respiratory syndrome coronavirus 2 (SARS-CoV-2) and SARS as well as MERS, such as that they are all mainly transmitted through respiratory droplets, and their most common symptom is fever [1–3]. Yet the SARS-CoV-2 was also found with R0 [4]. It has more clinical symptoms of dyspnoea while headaches and diarrhoea are rare [5]. As to radiological features, COVID-19 usually has abnormalities on chest computed tomography (CT), of which the most typical one is ground-glass opacity, and the lesions are generally multiple and located in the posterior or peripheral lung [6]. Patients infected with SARS-CoV-2 normally develop symptoms in 2−7 days after infection with a rapid disease progress. They usually die of acute respiratory distress syndrome (ARDS) and multiple organ failure caused by cytokine storm [5].

Despite many reports on SARS-CoV-2, few studies focused on disease progress and it is still unclear how clinical, radiological characteristics and laboratory indicators and their dynamic changes as disease progressed are related to prognosis. In this study, we comprehensively collected the clinical, radiological characteristics and laboratory information of 101 confirmed COVID-19 patients in Renmin Hospital of Wuhan University and aimed to explore the association between prognosis and the variation trend of those indicators.

## Methods

The study was approved by the Ethics Committee of the Wuhan University Renmin Hospital (ethics number: V1.0), and written informed consent was waived. A retrospective study was performed on 101 laboratory-confirmed patients with SARS-CoV-2, who were randomly selected from all the confirmed patients from 2 January 2020 to 28 January 2020, at Wuhan University Renmin Hospital, one of the first hospitals designated by the Chinese government to treat COVID-19, using isolation and airborne precautions (fit-tested N95 masks) for aerosol-generating immediately. The laboratory-confirmed patients were defined as positive results from any one of the following specimens: respiratory secretions obtained from bronchial alveolar lavage, tracheal aspirate, nasopharyngeal or oropharyngeal swabs. Clinical, imaging and laboratory data were collected and privacy masking was performed in data analysis. The follow-up endpoint was on 8 February 2020.

### Data collection

The demographic and epidemiological characteristics, laboratory findings as well as clinical diagnosis and treatment outcomes were extracted from electronic medical records. Several standardised data collection forms were designed first and relevant data were then correspondingly extracted by two experienced doctors and a third senior doctor would perform the final review to ensure data quality. Laboratory indicators of blood routine test, blood coagulation and their medical reference ranges were collected. The presence of SARS-CoV-2 in respiratory specimens was detected by real-time RT-PCR methods, issued by the Chinese CDC [7]. All the thin-layer images were collected in the format of Digital Imaging and Communications in Medicine (DICOM). Then the imaging signs and their features were extracted using a double-blind method, by which two intermediate radiologists in respiratory medicine (with more than 5 years' experience) would read and report the images independently and their results would finally be reviewed and confirmed by a senior doctor (engaged in imaging diagnosis for more than 15 years). Radiological findings such as lesions, lesion size (the longest diameter of the lesion) and radiological patterns were extracted. In addition, to evaluate the lesion size accurately, a diagnosis system for novel coronavirus pneumonia based on artificial intelligence (AI), was employed to analyse CT images using CT value to measure volume ratio of pneumonia automatically, which had been used at this hospital in early January [8–11].

### Clinical staging

The guidelines on COVID-19 treatment issued by the National Health Commission of the People's Republic of China have classified patients into four types, including uncomplicated illness, mild, severe and critical pneumonia [12]. Patients with uncomplicated illness have mild clinical symptoms but no signs of pneumonia on chest images, while mild pneumonia has fever and respiratory symptoms as well as pneumonitis signs on radiographs. Severe patients have at least one of the following: (1) respiratory distress, respiratory rate (RR)⩾30 breaths/min; (2) finger oxygen saturation⩽93% in a state of rest; (3) partial pressure of oxygen (PaO_2_)/fraction of inspired oxygen (FiO_2_) ⩽300 mmHg. Critical pneumonia has one of respiratory failure with mechanical ventilation required, shock, other organs failure required ICU monitoring. It should be noted that each patient may have multiple clinical stages as the disease progressed. We recorded all the clinical classifications for every selected patient and the time at which the classification began or change of classification occurred. The time (±2 days) was then used to match their latest imaging and laboratory information for each clinical stage of the patients.

### Statistical analysis

Clinical characteristics were described via means and standard deviations (for normally distributed variables) or interquartile range (Median(IQR)) (for not normally distributed variables) or frequency and percentages (for categorical variables), and their differences were then correspondingly compared by *t* test, Mann−Whitney *U* test (or Kruskal−Wallis *H* test), Chi-square test (or Fisher's exact probability), respectively. Cochran−Armitage trend test was further used to check whether an upward or downward trend exists in ordinary variables. A *P*-value <0.05 was considered statistically significant. All analyses were performed using SAS 9.4 (SAS Institute, Cary, NC, 2017) while graphs were plotted by R-3.6.0.

## Results

### Demographic and clinical characteristics of study participants

The median age of the 101 patients was 51.0 years (IQR 37.0–61.0), and 53 (52%) were female. Among them, 34 (34%) were medical staff and 47 (47%) had suspected nosocomial infections. More than half of the patients (51%) had coexisting disorders, which mainly were hypertension (20%), cardiovascular or cerebrovascular disease (14%), diabetes (13%) and malignant tumours (12%). None of the 101 patients had a history of exposure to the Wuhan Huanan seafood market. The common symptoms include fever (68%), cough (50%), fatigue (23%), myalgia (16%) and dyspnoea (14%). Mechanical ventilation, primarily nasal cannula/oxygen mask (20 patients, 20%), was used in 26 patients (26%). Almost all the patients (98%) were treated with antibiotics. While interferon, antiviral, gamma globulin and thymosin therapy were initiated in 40 (40%), 85 (84%), 65 (64%) and 10 (10%) patients, respectively. The median time from the initial symptom, hospital admission to the laboratory-confirmed diagnosis were 7.0 days (IQR: 5.0–13.0) and 4.0 days (IQR: 2.0–7.0). Overall, the person-times of uncomplicated illness, mild, severe and critical pneumonia was there were 1, 88, 55 and 14 during course of illness, respectively. As of 8 February 2020, more than one-third (37, 37%) were cured and discharged, one half (53, 52%) remained hospitalised, respectively, while 11 (11%) patients died unfortunately, as shown in Table 1.

**Table 1.** Clinical characteristics of 101 patients with COVID-19

Variables	Total (*N* = 101)	Non-medical staff (*N* = 67)	Medical staff (*N* = 34)	*P* value
Age, years	51.0 (37.0–61.0)	57.0 (44.0–64.0)	34.0 (30.0–42.0)	<0.001***
Sex				0.44
Male	48 (48%)	30 (45%)	18 (53%)	
Female	53 (52%)	37 (55%)	16 (47%)	
Suspected nosocomial infection	47 (47%)	13 (19%)	34 (100%)	<0.001***
Coexisting disorders	52 (51%)	48 (72%)	4 (12%)	<0.001***
Diabetes	13 (13%)	13 (19%)	0 (0%)	0.004^a^**
Hypertension	20 (20%)	19 (28%)	1 (3%)	0.002**
Cardiovascular or cerebrovascular disease	14 (14%)	14 (21%)	0 (0%)	0.002^a^**
Chronic obstructive pulmonary disease	2 (2%)	2 (3%)	0 (0%)	0.55^a^
Malignant tumours	12 (12%)	12 (18%)	0 (0%)	0.007^a^**
Chronic liver disease	6 (6%)	5 (7%)	1 (3%)	0.66^a^
Chronic renal disease	4 (4%)	4 (6%)	0 (0%)	0.30^a^
History of exposure to the Wuhan seafood market	0 (0%)	0 (0%)	0 (0%)	NA
Smoking	5 (5%)	4 (6%)	1 (3%)	0.66^a^
Pregnancy	1 (1%)	0 (0%)	1 (3%)	0.34^a^
Existing initial symptoms	90 (89%)	58 (87%)	32 (94%)	0.33^a^
Fever	69 (68%)	45 (67%)	24 (71%)	0.73
Cough	50 (50%)	27 (40%)	23 (68%)	0.009**
Expectoration	20 (20%)	14 (20%)	6 (18%)	0.70
Fatigue	23 (23%)	14 (21%)	9 (26%)	0.53
Chest distress	13 (13%)	9 (13%)	4 (12%)	1^a^
Vertigo	4 (4%)	4 (6%)	0 (0%)	0.30^a^
Myalgia	16 (16%)	7 (10%)	9 (26%)	0.037*
Headache	6 (6%)	3 (4%)	3 (9%)	0.40^a^
Diarrhoea	1 (1%)	0 (0%)	1 (3%)	0.34^a^
Dyspnoea	14 (14%)	14 (21%)	6 (18%)	0.002^a^**
Nausea	3 (3%)	3 (4%)	0 (0%)	0.55^a^
Time from initial symptoms to diagnosis	7.0 (5–13)	10.0 (6–15)	5.5 (2–7)	<0.001***
Time from admission to diagnosis	4.0 (2–7)	6.0 (3–10)	1.5 (0–3)	<0.001***
Physical examination on admission				
Temperature, °C	36.8 (36.5–37.2)	36.8 (36.5–37.5)	36.8 (36.6–37)	0.64
Pulse, mean (s.d.)	84.0 (13.0)	87.7 (12.6)	76.9 (10.5)	<0.001***
Respiratory rate	19.0 (18–20)	20.0 (19–20)	19.0 (18–19)	<0.001***
Systolic blood pressure, mm Hg	125.0 (117–133)	125.0 (113–138)	123.0 (118–125)	0.30
Diastolic blood pressure, mm Hg	75.0 (69–81)	76.0 (69–83)	75.0 (69–78)	0.220
Mechanical ventilation	26 (26%)	24 (36%)	2 (6%)	0.001**
Ventilatory type				0.600^a^
Nasal cannula/oxygen mask	20 (80%)	18 (78%)	2 (100%)	
Non-invasive ventilation or high-flow nasal cannula	3 (3%)	3 (13%)	0 (0%)	
Invasive mechanical ventilation	2 (2%)	2 (9%)	0 (0%)	
Drugs				
Glucocorticoid	66 (65%)	43 (64%)	23 (68%)	0.73
Antibiotic	99 (98%)	66 (99%)	33 (97%)	1^a^
Interferon	40 (40%)	10 (15%)	30 (88%)	<0.001***
Antiviral drug	85 (84%)	54 (81%)	31 (91%)	0.17
Gamma globulin	65 (64%)	43 (64%)	22 (65%)	0.96
Thymosin	10 (10%)	7 (10%)	3 (9%)	1^a^
Clinical stages				
Uncomplicated illness	1 (1%)	0 (0%)	1 (3%)	0.34^a^
Mild	88 (87%)	57 (85%)	31 (91%)	0.53^a^
Severe	55 (54%)	45 (67%)	10 (29%)	<0.001***
Critical	14 (14%)	14 (21%)	0 (0%)	0.002^a^**
Outcome				0.044*
Death	11 (11%)	11 (16%)	0 (0)	
Hospitalisation	53 (52%)	33 (49%)	20 (59%)	
Cured	37 (37%)	23 (34%)	14 (41%)	

*Note*: Data are presented as medians (interquartile ranges, IQR) and *N* (%).

a *P* value was calculated using Fisher's exact probability instead of *χ*^2^ test.

*P value < 0.05, **P value < 0.01, ***P value < 0.001.

**Fig. 1.**
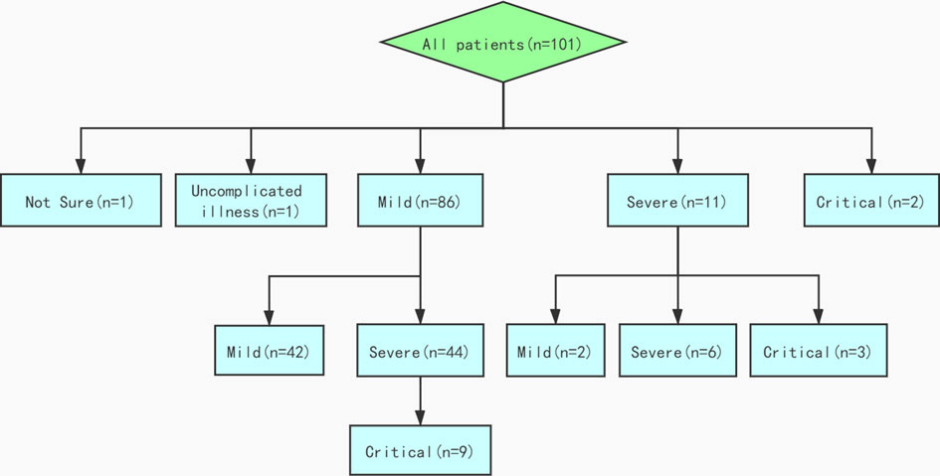
Flowchart of disease progression of 101 patients.

Figure 1 depicted the flowchart of disease progress of 101 patients. On admission, 1, 86, 11 and 2 of the 101 patients were diagnosed as uncomplicated illness, mild, severe, and critical pneumonia, respectively. Additionally, one woman did not have a clinical stage due to pregnancy. During the course of illness, 44 (51%) mild patients progressed to severe condition within a median time of 4 days (IQR: 1–7), among which nine cases (10%) further worsened to critical pneumonia within 4 days (IQR: 3–8) from severe illness. While two of the 11 severe patients improved to mild condition, three others deteriorated to critical condition.

### Radiological characteristics stratified by clinical stages

**Fig. 2.**
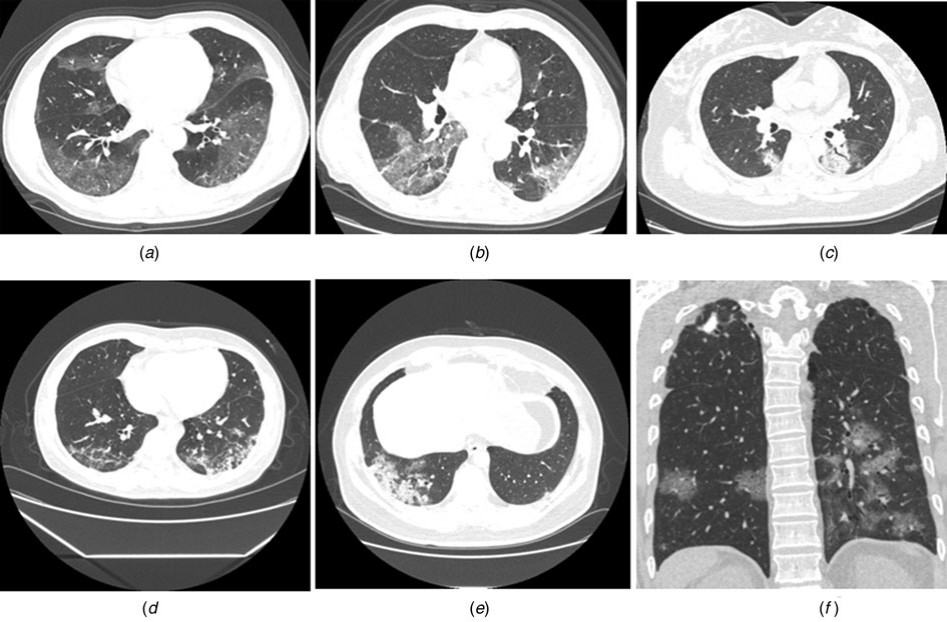
Typical radiological patterns on chest CT images. (a) pure ground-glass opacity; (b) round-glass opacities overlapped with striped shadows; (c) pulmonary consolidation; (d) reticular patterns; (e) mixed patterns and (f) others. The illustration depicted a secondary tuberculosis case infected with SARS-CoV-2.

Radiological abnormalities were found in 121 CT scans. The majority of abnormalities were presented with multiple lesions (105, 87%), bilateral lungs affected (97, 80%). The most common lesions located in the posterior basal segment of the left lower lobe (LLL) (72%), followed by the posterior basal segment of the right lower lobe (RLL) (67%), while the anterior basal segment of the RLL and the anterior segment of the left upper lobe (LUL) were affected the least (36% and 40%). The most common patterns on chest CT were pure ground-glass opacity ((44, 36%), Fig. 2a) and ground-glass opacities overlapped with striped shadows ((48, 40%), Fig. 2b). In addition, 18 cases showed pulmonary consolidation (Fig. 2c), four showed reticular patterns (Fig. 2d) and three mixed patterns (Fig. 2e). Most of the cases (65%) were accompanied by peripheral vessels thickening, followed by air bronchogram (44%), mosaic signs (14%), pleural effusion (14%) and halo signs (13%). Significant differences were observed in bilateral/left/right lungs of being influenced (*P* = 0.07), multiple or single lesions (*P* = 0.033), lesion size (*P* = 0.003), numbers of influenced pulmonary segments (*P* < 0.001) and numbers of pulmonary segments with striped shadows (*P* = 0.017) among groups with different clinical stages (Table 2).

**Table 2.** Radiological findings stratified by clinical stages

Variables	Overall (*N* = 121)	Mild (*N* = 70)	Severe (*N* = 42)	Critical (*N* = 9)	*P* value
Radiological abnormalities					0.75^a^
Ground-glass opacities overlapped with striped shadows	48 (40%)	31 (44%)	14 (33%)	3 (33%)	
Pure ground-glass opacity	44 (36%)	25 (36%)	15 (36%)	4 (44%)	
Mixed patterns	3 (2%)	1 (1%)	2 (5%)	0 (0)	
Consolidation	18 (15%)	10 (14%)	7 (17%)	1 (11%)	
Reticular patterns	4 (3%)	2 (3%)	2 (5%)	0 (0%)	
Others	4 (3%)	1 (1%)	2 (5%)	1 (11%)	
Bilateral lungs affected					0.07^a^
Bilateral lungs	97 (80%)	50 (71%)	38 (91%)	9 (100%)	
Right lung	12 (10%)	11 (16%)	1 (2%)	0 (0%)	
Left lung	12 (10%)	9 (13%)	3 (7%)	0 (0%)	
Single or multiple lesions^a^					0.033*
Single lesion	16 (13%)	14 (20%)	2 (5%)	0 (0%)	
Multiple lesions	105 (87%)	56 (80%)	40 (95%)	9 (100%)	
Lesion size					0.003^a^**
<1 cm	4 (3%)	3 (4%)	1 (2%)	0 (0%)	
1–3 cm	35 (29%)	23 (33%)	11 (26%)	1 (11%)	
3 cm-50% lung lobe	66 (55%)	42 (60%)	20 (48%)	4 (44%)	
>50% lung lobe	16 (13%)	2 (3%)	10 (24%)	4 (44%)	
AI-based volume ratio of pneumonia					
−700~500	0.19 (0.11–0.29)	0.16 (0.10–0.26)	0.20 (0.14–0.35)	0.49 (0.32–0.54)	0.002
−600~500	0.12 (0.07–0.20)	0.09 (0.07–0.17)	0.14 (0.09–0.25)	0.32 (0.20–0.41)	0.002
−500~−200	0.05 (0.03–0.09)	0.04 (0.03–0.08)	0.07 (0.04–0.11)	0.14 (0.09–0.20)	0.003
Number of affected lung segments	9.5 (3–14)	6.0 (2–12)	12.0 (6–15)	17.0 (10–18)	<0.001***
Air bronchogram	53 (44%)	25 (36%)	21 (50%)	7 (78%)	0.034*
Vessel thickening	79 (65%)	42 (60%)	30 (71%)	7 (78%)	0.34
Halo signs	16 (13%)	8 (11%)	7 (17%)	1 (11%)	0.69
Reverse halo signs	5 (4%)	2 (3%)	3 (7%)	0 (0%)	0.57^a^
Mosaic signs	17 (14%)	5 (7%)	9 (21%)	3 (33%)	0.024*
Emphysema	16 (13%)	7 (10%)	7 (17%)	2 (22%)	0.42
Pleural effusion	17 (14%)	5 (7%)	10 (24%)	2 (22%)	0.037*
Striped shadows	72 (60%)	40 (57%)	27 (64%)	5 (56%)	0.73
Number of lung segments with striped shadows	7.0 (3–12)	4.0 (2–8)	9.5 (5–13)	9.5 (8–13.5)	0.017*
Number of lung segments with abnormalities					
Apical segment of RULL	59 (49%)	27 (46%)	26 (44%)	6 (10%)	0.028^a^*
Posterior segment of RULL	66 (55%)	31 (47%)	28 (42%)	7 (11%)	0.029^a^*
Anterior segment of RULL	46 (38%)	21 (46%)	20 (43%)	5 (11%)	0.094
Outer segment of RMLL	62 (51%)	31 (50%)	24 (39%)	7 (11%)	0.12^a^
Inner segment of RMLL	50 (41%)	24 (48%)	21 (42%)	5 (10%)	0.18
Dorsal segment of RLLL	69 (57%)	35 (50%)	29 (42%)	5 (7%)	0.14
Basal segment of RLLL	57 (47%)	23 (40%)	26 (46%)	8 (14%)	<0.001^a^***
Anterior basal segment of RLLL	43 (36%)	15 (35%)	22 (51%)	6 (14%)	<0.001***
Outer basal segment of RLLL	74 (61%)	41 (55%)	27 (36%)	6 (8%)	0.78
Posterior basal segment of RLLL	83 (69%)	44 (53%)	31 (37%)	8 (10%)	0.19
Posterior apical segment of LULL	49 (41%)	23 (47%)	21 (43%)	5 (10%)	0.13
Anterior segment of LULL	48 (40%)	23 (48%)	20 (42%)	5 (10%)	0.18
Upper lingual segment of LULL	50 (41%)	21 (42%)	22 (44%)	7 (14%)	0.005**
Sublingual segment of LULL	62 (51%)	27 (44%)	28 (45%)	7 (11%)	0.004^a^**
Dorsal segment of LLLL	68 (56%)	31 (46%)	31 (46%)	6 (9%)	0.008***
Anterior medial basal segment of LLLL	61 (50%)	29 (48%)	25 (41%)	7 (11%)	0.045^a^*
Outer basal segment of LLLL	70 (58%)	34 (49%)	29 (41%)	7 (10%)	0.047*
Posterior basal segment of LLLL	87 (72%)	42 (48%)	37 (43%)	8 (9%)	0.003**

RULL, right upper lung lobe; RLLL, right lower lung lobe; RMLL, right middle lung lobe; LULL, left upper lung lobe; LLLL, left lower lung lobe.

*Note*: Data are presented as medians (interquartile ranges, IQR) and *N* (%).

a *P* value was calculated using Fisher's exact probability instead of *χ*^2^ test.

*P value < 0.05, **P value < 0.01, ***P value < 0.001.

### Radiological findings as disease progressed

The average interval between the second, third and fourth CT examinations and the first time was 4, 7 and 10 days, respectively. Besides, single lesion gradually developed to multiple ones in the disease progression, with an increasing trend (*P* = 0.05) in median affected pulmonary segments (7.0, 9.0, 10.5 and 12.0) (Table S1 in Supplementary Appendix). In terms of radiological patterns, an upward trend was observed in the numbers of ground-glass opacities overlapped with striped shadows (33%, 42%, 55% and 80%), as shown in Table S1. Correspondingly, the percentage of pure ground-glass opacities presented with a decreasing trend (45%, 35%, 24% and 13%) for the first time CT scans to the fourth time CT scans within 12 days as the disease progressed. Representative radiological findings during course of illness could be found in Figure S1.

### Laboratory parameters stratified by clinical stages

**Fig. 3.**
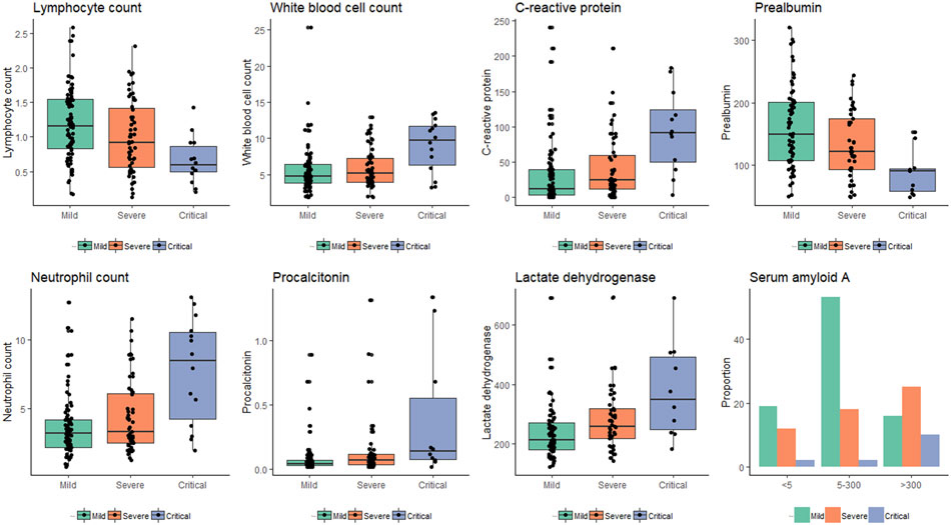
Laboratory parameters stratified by clinical stages.

Significant differences were observed among groups of different clinical stages in lactate dehydrogenase (*P* = 0.001), blood urea (*P* = 0.03), procalcitonin (*P* = 0.002) and D-dimer (*P* < 0.001), prothrombin time (*P* = 0.003), lymphocyte count (*P* = 0.001), white blood cells (*P* = 0.006), neutrophil count (*P* = 0.001), lymphocyte ratio (*P* < 0.001), C-reactive protein (*P* < 0.001), albumin (*P* < 0.001), prealbumin (*P* < 0.001) and serum amyloid (*P* < 0.001) (Table 3). As illustrated in Figure 3, lymphocytes prealbumin and albumin decreased as disease progressed while an upward trend was witnessed in white blood cells, C-reactive protein, neutrophils and lactate dehydrogenase decrease over time. The most predominant spectrum of serum amyloid A was 5−300, >300 and >300 mg/l in mild (60%), severe (46%), critical (71%) conditions, respectively.

**Table 3.** Laboratory parameters stratified by clinical stages

Parameters	Total	Patients (*N* = 101)			*P*-value
		Mild (*N* = 88)	Severe (*N* = 55)	Critical (*N* = 14)	
Creatine kinase U/l	68.5 (41.2–100.5)	73.0 (48.5–97.0)	64.0 (33.0–103.0)	53.5 (37.3–93.3)	0.70
Lactate dehydrogenase U/l	237.0 (191.5–302.5)	213.0 (179.5–271.0)	257.0 (217.0–319.0)	350.0 (248.0–494.0)	0.001**
ALT U/l	21.0 (14.0–33.0)	19.5 (13.0–31.5)	21.5 (15.3–38.5)	25.0 (19.8–30.3)	0.42
AST U/l	24.0 (19.0–32.3)	24.0 (19.0–30.0)	24.5 (19.0–32.0)	35.0 (21.8–42.8)	0.16
Total bilirubin μmol/l	8.9 (7.4–12.8)	8.6 (7.4–12.8)	8.7 (7.3–12.8)	11.0 (8.8–12.5)	0.78
Creatinine mmol/l	59.0 (50.0–81.3)	60.5 (51.0–77.8)	56.0 (48.0–84.5)	62.0 (50.5–112.3)	0.71
Blood urea mmol/l	4.5 (3.7–6.0)	4.3 (3.7–5.1)	4.8 (3.1–6.7)	6.4 (5.1–10.2)	0.03*
Procalcitonin ng/ml	0.06 (0.03–0.12)	0.04 (0.03–0.09)	0.08 (0.04–0.13)	0.17 (0.09–1.26)	0.002**
D-dimer mg/l	0.5 (0.2–1.3)	0.4 (0.2–0.7)	0.6 (0.3–1.2)	1.7 (1.0–3.4)	<0.001***
Prothrombin time sec	11.3 (10.9–12.0)	11.2 (10.7–11.8)	11.3 (10.9–11.8)	12.1 (11.7–12.9)	0.003
Platelet count 10^9^/l	163.5 (122.3–213.8)	161 (128.0–195.0)	180.0 (122.5–237.5)	165.5 (107.5–229.0)	0.54
Monocyte count 10^9^/l	0.4 (0.3–0.6)	0.5 (0.3–0.6)	0.4 (0.2–0.5)	0.3 (0.1–0.6)	0.12
Lymphocyte count 10^9^/l	1.0 (0.7–1.5)	1.15 (0.8–1.5)	0.92 (0.6–1.4)	0.59 (0.5–0.9)	0.001***
White blood cells 10^9^/l	5.2 (3.9–7.1)	4.8 (3.8–6.4)	5.2 (4.0–7.3)	9.8 (6.3–11.7)	0.006**
Neutrophil count 10^9^/l	3.3 (2.4–5.2)	3.2 (2.2–4.2)	3.3 (2.5–6.1)	8.5 (4.2–10.6)	0.001**
Lymphocyte ratio %	21.7 (12.3–30.5)	24.6 (15.9–32.4)	18.0 (8.7–28.9)	5.5 (4.3–14.9)	<0.001***
CRP mg/l	17.2 (5.9–56.0)	12.6 (3.9–39.6)	24.7 (12.0–59.8)	91.6 (49.8–124.6)	<0.001***
Albumin g/l	37.0 (33.7–40.8)	39.6 (36.5–42.1)	34.7 (32.6–37.8)	31.9 (29.9–36.0)	<0.001***
Direct bilirubin μmol/l	3.4 (2.5–4.8)	3.3 (2.5–4.3)	3.3 (2.6–5.0)	4.2 (3.3–5.5)	0.36
Globulin g/l	23.4 (21.6–26.8)	23.3 (21.2–25.5)	24.09 (21.8–27.2)	26.8 (24.2–28.7)	0.06
Prealbumin mg/l	130.8 (95.1–189.1)	149.6 (107.5–200.8)	121.59 (92.22–174.7)	89.9 (57.3–94.3)	<0.001***
Serum amyloid A mg/l					<0.001***
<5.0	33	19 (22%)	12 (22%)	2 (14%)	
5.0–300.0	73	53 (60%)	18 (33%)	2 (14%)	
>300.0	51	16 (18%)	25 (46%)	10 (71%)	

*Note*: Data are presented as medians (interquartile ranges, IQR) and *N* (%).

*P value < 0.05, ** *P* value < 0.01, *** *P* value < 0.001.

## Discussion

This retrospective study, to our best knowledge, is the first report to the disease progression of patients with SARA-CoV-2. As of 28 January 2020, a total of 101 confirmed COVID-19 patients were enrolled in this study, and classified into four different groups (uncomplicated illness, mild, severe and critical pneumonia) according to the disease severity. Among these patients, 86 and 11 patients were diagnosed as mild and severe pneumonia, as well as one uncomplicated illness and two critical pneumonia on admission. The median age of these patients was 51.0 years (IQR 37.0–61.0), 34% were medical staff and 47% has suspected nosocomial infections, this is caused by the rapid person-to-person transmission of COVID-19 with less personal protection. Fever (68%), cough (50%), fatigue (23%) and myalgia (16%) are the most symptoms on admission. Fifty-one per cent patients have complications, with hypertension (20%), cardiovascular or cerebrovascular disease (14%) and diabetes (12%), respectively. The most common patterns on chest CT were pure ground-glass opacity (36%) and ground-glass opacities overlapped with striped shadows (40%). About 26% patients underwent the mechanical ventilation. These findings conform with the previous study of COVID-19 in Wuhan, China [5, 13]. As of 8 February 2020, more than one-third (37, 37%) of patients were cured and discharged, a half (53, 52%) were still hospitalised, while 11 (11%) patients died unfortunately. The median time from initial symptoms to diagnosis was 7 days (IQR 5–13), as well as 4.0 days (IQR 2–7) for median time from admission to diagnosis. This finding further confirms the viewpoint that the median time from onset to admission was 7 days by Huang *et al*. [5]. However, the median time from initial symptoms to diagnosis was shortened greatly during January (Figure S2), this is due to the substantial supplies of SARS-Cov-2 detection kit. This sharp downtrend could also be witnessed for median time from admission to diagnosis.

Notable, the majority (72%) of the non-medical-staff patients had complications, which primarily were cardiovascular diseases and diabetes. This proportion was higher than SARS but close to MERS [14], suggesting that patients with pre-existing conditions should get well-prepared for the prevention of SARS-CoV-2. On admission, most patients had fever (68%), the most common symptom of the disease in the study. Importantly, a significant proportion of patients without fever in an early stage, and were more vulnerable to be ignored, indicating that more attention should be given to patients without fever when screening and diagnosing COVID-19.

Additionally, 86 of the 101 patients were diagnosed with mild pneumonia on admission, 42 of the 86 patients maintained or improved. Meanwhile, 19 of 42 get cured in a short period and no death occurred. While, 44 of the 86 mild pneumonia patients progressed to severe condition within 4 days, among which 13 were cured and 11 died. This suggested that we should pay more attention to the disease at an early stage, rather than neglecting the mild illness. Besides, we could see that COVID-19 progresses rapidly within 4 days from mild-to-severe condition, further illustrated by variations in radiological characteristics over time in this study. Indeed, it is significantly crucial to speed up the diagnosis process of suspected cases and make sure all potential patients are able to get access to hospitals for observation and treatment at an early stage. Furthermore, patients with complications could highly develop severe and critical illness as disease progressed. Also whether or not having dyspnoea as initial symptoms and the respiratory frequency in physical examination on admission were both significantly different among groups of different clinical stages (higher probability observed in severe or critical stage than the mild one), suggesting that attention should be paid to the patient's underlying disease and first symptoms when treating COVID-19 [8, 15–17].

Moreover, most patients presented with patchy, lumpy or segmental ground-glass shadows in the subpleural area in an early stage. Also, the most common lesions were on the posterior and lateral sides of the lower pulmonary lobe. Yet it was light in the shadows of infiltration of inflammation in lung lobe at the beginning, mainly related to its pathological changes, still suggesting that the SARS-CoV-2 already affected the lung before symptoms were being recognised. As the disease progressed, an upward trend was observed in the numbers of ground-glass opacities overlapped with striped shadows, of affected pulmonary segments, illustrating that pulmonary fibrosis became more severe and to some extent further suggesting that patients are at risk of irreversible damage to lung function in the future, which may further lead to functional disability and affect the quality of life. Such sequelae occurred in both patients infected with SARS or MERS [11, 18]. Hence, adding supportive treatment to the therapeutic regimen should be taken into consideration to improve patient's immunity, to weaken the damages to the lungs caused by viral pneumonia and to reduce the risk of sequelae. In addition, no significant difference was shown in typical radiological patterns among groups of different clinical stages but mosaic signs and pleural effusion, which might relate to prognosis. Meanwhile, AI-based diagnosis system showed similar results in assessment of lesions size as what was evaluated by the radiologists. It is also worth mentioning that such AI system greatly improved diagnostic efficiency of COVID-19, alleviating the surge in demand of radiologists when the virus is stalking China.

In this study, significant differences were found in lactate dehydrogenase, D-dimer, prothrombin, lymphocyte count, white blood cell count, neutrophil count, C-reactive protein, albumin and serum amyloid A as the disease progressed. Some of those indicators were inconsistent with previous studies [13]. In addition, we observed that the amount of albumin and prealbumin in critically or severely ill patients were significantly lower than those of mild ones. Such differences might relate to the cytokine storm caused by the virus at the late stage of the disease and other factors, such as subsequent hypoxia, gastrointestinal dysfunction, endocrine and metabolic disorders, insufficient nutrition intake and the use of ventilator with positive pressure, and increased energy consumption in the body. Eventually, malnutrition would be caused or aggravated. As a negative acute phase response protein and a kind of substance in non-specific host defense, prealbumin can clear the toxic metabolites released in the circulation during the infection process, and therefore its amount reduced in blood as it was gradually consumed. This reminds us to provide nutrition support for these kind of patients [19]. Meanwhile, we found that some inflammation indicators, such as C-reactive protein, and serum amyloid A, increased in the early stage of the disease, but higher amounts of them were observed in severe and critical patients than that of mild cases. This might indicate the patients were in the state of acute stress with elevated serum amyloid A and C-reactive protein, which are able to bind various microorganisms' polysaccharides and tissue cells' phospholipids and nucleic acids to activate the immune system, improving immunity and mediating inflammatory responses.

No difference on treatment effect of various drugs was observed for patients with different disease progression states; it might be due to the fact that there is no specific treatment guideline for patients with novel coronavirus pneumonia at different states, which might be the main causes for the death of severe and critical patients. In addition, the evidences collected in this study cannot confirm which treatment can obviously control the disease progression among current clinical treatment regimens. Therefore, pro-active actions should be taken to accelerate the clinical trial for potential effect drugs, such as Remdesivir, and various drug combinations [9, 20]; meanwhile, more existing drugs on the market, which can enhance immune response, reduce immunopathology and prevent or suppress ARDS, such as metformin, glitazone, sartans, atorvastin and relative nutritional supplements and biologics, might be effective treatment options for this disease [4, 21–23].

This study has several limitations. Firstly, only 48 of the 101 patients included in the study currently have an endpoint, and the others are still under treatment, therefore no indicators related to prognosis could be selected and further explored. Secondly, despite the fact that we believed that cellular and humoral immunity play an important role in the disease process, it was not used as a routine test, as we did not fully understand the disease at an early stage, making these important indicators impossible to analyse. Nevertheless, we were to collect relevant information for further analysis. Thirdly, data samples are limited because we need to continually and actively fight and treat SARS-CoV-2.

## Conclusion

The COVID-2019 progresses rapidly and early intervention and treatment are critically crucial for patients' prognosis. Radiological patterns and laboratory parameters can timely reflect disease's progression, providing useful help for disease's diagnosis and treatment.

## Supplementary material

For supplementary material accompanying this paper visit http://dx.doi.org/10.1017/S0950268820000977. click here to view supplementary material
