# Supplementary material for: Characteristics of disease progress in patients with coronavirus disease 2019 in Wuhan, China
Source: Epidemiol Infect. 2020 May 6;148:e94. doi: 10.1017/S0950268820000977 (PMC7225215; doi:10.1017/S0950268820000977)
Supplement: Supplementary file 1 [file S0950268820000977sup001.docx]

**Supplementary Appendix**

**Table S1**. Radiologic findings as disease progressed.

**Figure S1.** Radiological findings in the course of illness. a～c were one patient’s chest images of the 3rd, 8th and 16th days from when SARS-CoV-2 was laboratory-confirmed; and d～f illustrated the chest images of the 2nd, 7th, and 12th days of another confirmed case. a) multiple lesions with ground-grass opacities in bilateral lungs without other accompanied patterns; b) the size of lesions in bilateral lungs was slightly enlarged with ground-grass opacities overlapped with fibrous striped shadows; c) the density of lesions in the lower lobe of both lungs was lightened with an absorption-like change; d): ground-glass like shadows distributed along pulmonary segments (mainly in the upper right lobe), accompanied by air bronchial and vascular thickening patterns; e): increased ranges of lesions in the right upper lobe, novel lesions observed in the left lobe with air bronchogram and vessels thickening, plus a small amount of fluid in the right pleural cavity; f) mixed opacities effused to the whole lungs, accompanied by air bronchial and vascular thickening patterns plus bilateral pleural effusion, known as "white lung".

**Figure S2**. Time from initial symptoms or admission to diagnosis in different period.

Table S1. Radiologic findings as disease progressed

| **Variables** | **1^st^ time (N=95)** | **2^nd^ time (N=71)** | **3^rd^ time (N=42)** | **4^th^ time (N=15)** | ***P*** |
| --- | --- | --- | --- | --- | --- |
| Time from the 1^st^ chest examination (days) | 0(0-0) | 3(2-4) | 7(6-8) | 10(8-11.5) | <0.001 |
| Radiological abnormalities ^a^ |  |  |  |  | <0.001 |
| Ground-glass opacities overlapped with striped shadows | 31(33%) | 30(42%) | 23(55%) | 12(80%) |  |
| Pure ground-glass opacity | 43(45%) | 25(35%) | 10(24%) | 2(13%) |  |
| Mixed patterns | 2(2%) | 2(3%) | 0(0%) | 0(0%) |  |
| Consolidation | 13(14%) | 10(14%) | 6(14%) | 1(7%) |  |
| Reticular patterns | 2(2%) | 2(3%) | 2(5%) | 0(0%) |  |
| Others | 4(4%) | 2(3%) | 1(2%) | 0(0%) |  |
| Bilateral lungs affected ^a^ | 70(74%) | 59(83%) | 37(88%) | 13(87%) | 0.010 |
| Single or multiple lesions ^a^ |  |  |  |  | 0.05 |
| Single lesion | 20(21%) | 5(7%) | 3(7%) | 1(7%) |  |
| Multiple lesions | 75(79%) | 66(93%) | 39(93%) | 14(93%) |  |
| Lesion size |  |  |  |  | 0.05 |
| < 1cm | 3(3%) | 3(4%) | 3(7%) | 0(0%) |  |
| 1-3cm | 40(42%) | 16(23%) | 9(21%) | 5(33%) |  |
| 3cm-50% lung lobe | 45(47%) | 40(56%) | 24(57%) | 7(47)% |  |
| > 50% lung lobe | 7(7%) | 12(17%) | 6(14%) | 3(20%) |  |
| AI-based volume ratio of pneumonia |  |  |  |  |  |
| -700~500 | 0.18(0.10-0.29) | 0.17(0.11-0.31) | 0.18(0.13-0.29) | 0.21(0.12-0.30) | 0.80 |
| -600~500 | 0.11(0.07-0.19) | 0.11(0.07-0.20) | 0.11(0.08-0.16) | 0.13(0.07-0.20) | 0.85 |
| -500~-200 | 0.04(0.03-0.09) | 0.05(0.03-0.09) | 0.05(0.04-0.07) | 0.06(0.03-0.09) | 0.68 |
| Air bronchogram ^a^ | 33(35%) | 31(44%) | 16(38%) | 5(33%) | 0.79 |
| Vessels thickening ^a^ | 56(59%) | 47(66%) | 25(60%) | 10(6%) | 0.63 |
| Halo signs ^a^ | 12(13%) | 8(11%) | 4(10%) | 1(7%) | 0.42 |
| Reverse halo signs ^a^ | 4(4%) | 2(3%) | 1(2%) | 0(0%) | 0.35 |
| Mosaic signs ^a^ | 9(9%) | 14(20%) | 5(12%) | 2(13%) | 0.52 |
| Emphysema ^a^ | 13(14%) | 9(13%) | 1(2%) | 1(7%) | 0.08 |
| Pleural effusion ^a^ | 7(7%) | 12(17%) | 5(12%) | 3(20%) | 0.14 |
| Striped shadows ^a^ | 51(54%) | 47(66%) | 30(71%) | 10(6%) | 0.05 |
| No. of affected lung segments ^a^ | 7.0(2-14) | 9.0(3-14) | 10.5(4-16) | 12.0(6-16) | 0.14 |

Note：Data are presented as medians (interquartile ranges, IQR) and N (%); **P* value<0.05, ** *P* value<0.01, *** *P* value<0.001;

^a^ denotes Cochran-Armitage trend test was further used to check whether an upward or downward trend exists, and significant trends (*P*<0.05) were found in radiological abnormalities (Ground-glass opacities overlapped with striped shadows vs All others), bilateral lungs affected and multiple lesions.


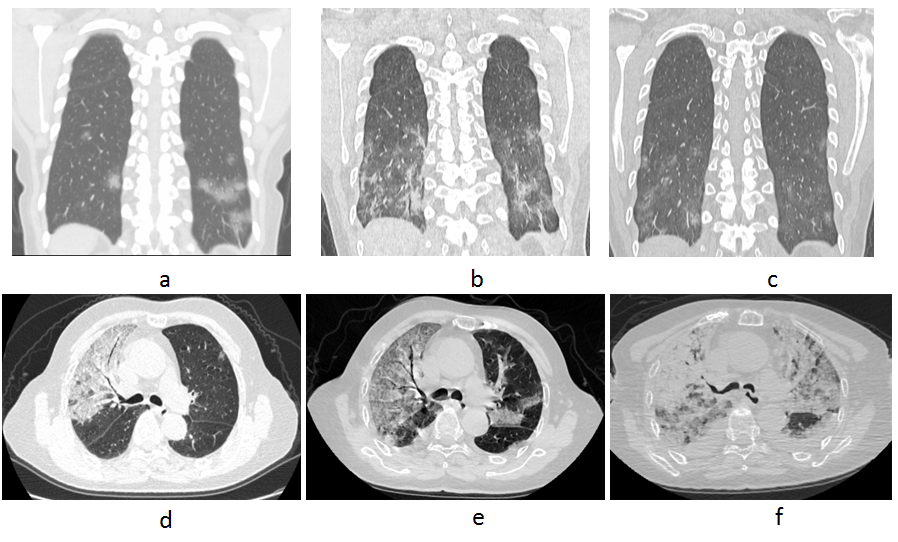


Figure S1: Radiological findings in the course of illness. a～c were one patient’s chest images of the 3rd, 8th and 16th days from when SARS-CoV-2 was laboratory-confirmed; and d～f illustrated the chest images of the 2nd, 7th, and 12th days of another confirmed case. a) multiple lesions with ground-grass opacities in bilateral lungs without other accompanied patterns; b) the size of lesions in bilateral lungs was slightly enlarged with ground-grass opacities overlapped with fibrous striped shadows; c) the density of lesions in the lower lobe of both lungs was lightened with an absorption-like change; d): ground-glass like shadows distributed along pulmonary segments (mainly in the upper right lobe), accompanied by air bronchial and vascular thickening patterns; e): increased ranges of lesions in the right upper lobe, novel lesions observed in the left lobe with air bronchogram and vessels thickening, plus a small amount of fluid in the right pleural cavity; f) mixed opacities effused to the whole lungs, accompanied by air bronchial and vascular thickening patterns plus bilateral pleural effusion, known as "white lung".


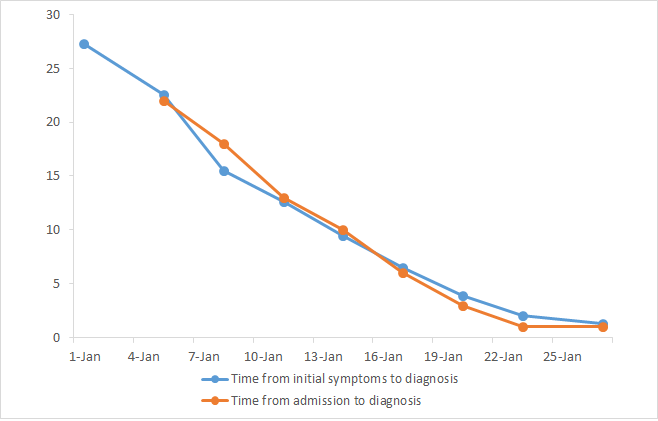


Figure S2. Time from initial symptoms or admission to diagnosis in different period.
